# Remediation of cognitive and motor functions in Tunisian elderly patients with mild Alzheimer’s disease: implications of music therapy and/or physical rehabilitation

**DOI:** 10.3389/fnagi.2023.1216052

**Published:** 2023-07-19

**Authors:** Sarah Chéour, Chouaieb Chéour, Tommy Gendreau, Majdi Bouazizi, Kumar Purnendu Singh, Ayoub Saeidi, Dan Tao, Rashmi Supriya, Nicola Luigi Bragazzi, Julien S. Baker, Foued Chéour

**Affiliations:** ^1^High Institute of Sport and Physical Education of Ksar-Saïd, Manouba, Tunisia; ^2^Physical Education and Sports Pavilion, Laval University, Quebec City, QC, Canada; ^3^High Institute of Sport and Physical Education of Gafsa, Gafsa, Tunisia; ^4^FEBT, School of Environment, Resources and Development, Asian Institute of Technology, Klong Luang, Pathum Thani, Thailand; ^5^Department of Physical Education and Sports Sciences, University of Kurdistan, Sanandaj, Iran; ^6^Department of Government and International Studies, Hong Kong Baptist University, Kowloon Tong, Hong Kong SAR, China; ^7^Centre for Health and Exercise Science Research, SPEH, Hong Kong Baptist University, Kowloon Tong, Hong Kong SAR, China; ^8^Laboratory for Industrial and Applied Mathematics (LIAM), Department of Mathematics and Statistics, York University, Toronto, ON, Canada; ^9^High Institute of Education and Continuous Training of Tunis, Tunis, Tunisia

**Keywords:** Alzheimer’s disease, music therapy, physical exercise, cognitive functions, motor functions, correlation, elderly patient

## Abstract

The purpose of this study was to compare the effects of music therapy (MT) and/or physical rehabilitation (PR) on cognitive and motor function in elderly Tunisian male and female patients with mild Alzheimer’s disease (AD). Male patients (*N*: 16; age: 74.19 ± 4.27 years; weight: 76.71 ± 5.22 kg) and female patients (*N*: 12; age: 71.46 ± 3.36 years; weight: 67.47 ± 4.31 kg) with mild AD were randomly assigned into 4 groups including control group (Co), PR group participated in physical rehabilitation, MT group received music therapy and MT + PR received both music therapy and physical rehabilitation. Participants were required to engage in the study for four months with three 60-min sessions per week. We found all scores of cognitive (MMSE, ADAS-Cog Total and the ADAS-Cog Memory subscale) and motor functions (step length, walking speed, 6MVT and BBS score) evaluated were the greatest in MT + PR compared to the other groups. Our study also demonstrated that MT has a greater effect on cognitive function, while PR has a more pronounced effect on motor function. Changes in MMSE scores were significantly positively correlated in the PR, MT and MT + PR groups with improvements in all motor functions including step length (*r* = 0.77), walking speed (*r* = 0.73), 6MVT (*r* = 0.75) and BBS scores (*r* = 0.78) in AD patients. In conclusion, the combination of MT and PR seems to be an appropriate intervention approach that needs consideration as a treatment strategy for elderly male and female patients with mild AD.

## Introduction

Recent research has reported that the global population has increased in age over the past century due to increased life expectancy. This has been partly attributed to improved health care and declining fertility rates (United Nations [UN], 2020). Indeed, aging induces changes in the levels of organs, tissues, and cells of the body. Histological studies have shown that aging induces severe neuroanatomical alterations in the central nervous system, causing an overall reduction in brain activity (Grothe et al., 2017; Xia et al., 2018). This damage to the central nervous system ultimately reduces control and coordination of essential body functions, and cognitive ability (Manini et al., 2013; NIH and Medline Plus, 2023). The physiological characteristics of Alzheimer’s disease have been linked to severe degeneration of cerebral neurons and alterations in synapses, particularly in the temporal, parietal, and frontal cortices. The degeneration observed induce progressive learning and memory deficits, cognitive decline and visuospatial disorientation (Rygiel, 2016; Grothe et al., 2017; Calabrò et al., 2021). These alterations are accompanied by emotional disturbances and loss of self-confidence, and lead to anxiety and depression in Alzheimer’s patients. This affects their social functioning and activities of daily living (Kravitz et al., 2012). The underlying mechanisms of AD remain unclear but are thought to be related to the abnormal build-up of proteins in and around brain cells. One of the proteins, β-amyloid, has been associated with the formation of plaques around brain cells. The other protein tau protein has been implicated in the formation of tangles within the brain causing neuronal degeneration combined with synaptic deficits (Calabrò et al., 2021). The causes of AD probably include a combination of age-related changes in the brain, along with genetic, environmental, and lifestyle factors. The importance of any one of these factors for increasing or decreasing the risk of AD is unclear, and may differ from person to person (Calabrò et al., 2021; Kocagoncu et al., 2022).

Pharmacological treatments appear to be unsatisfactory with minimal benefits for patients with AD. Indeed, drugs used to control this disease, were unable to decrease or stop neuronal damage and destruction or the progression of this pathology (O’Brien et al., 2017). In recent years, several scientific sources have established the beneficial effects of non-pharmacological therapies, mainly physical exercise and MT. These have been effective in improving the regenerative power of the brain and its plasticity, and consequently the cognitive and motor function of patients with Alzheimer’s (Fukui et al., 2012). The results of a prospective cohort study indicated that exercise was one of the healthy lifestyle factors that contribute positively to attenuating the risk of AD (Nuzum et al., 2020). There is now strong evidence linking regular physical activity to higher cognitive and motor function in Alzheimer’s patients. Exercise may also play a role in increasing peripheral blood concentrations in the brain and the secretion of neurotrophic factors which further reduce the risk of developing AD (Marinus et al., 2019). MT is also a common non-pharmaceutical treatment for AD (Eftychios et al., 2021). The effectiveness of MT can depend on the quality and length of treatment as well as other factor including type of music or state of mood (depressed or cheerful) (Leubner and Hinterberger, 2017). MT improves behaviors such as interpersonal interactions and social conversations by reducing wandering and restlessness. MT can stimulate almost any area of the brain, making the individual more conscious and aware, encourages the recall of vivid memories, and has the potential to lighten mood (Moreno-Morales et al., 2020; Eftychios et al., 2021).

Therefore, the objectives of this study were to evaluate the effectiveness of MT and PR used as a single or combined intervention on the improvement of cognitive and motor functions in elderly Tunisian male and female patients.

## Materials and methods

### Participants

Participants were 28 AD patients, including males (*N*: 16; age: 74.19 ± 4.27 years; weight: 76.71 ± 5.22 kg) and females (*N*: 12; age: 71.46 ± 3.36 years; weight: 67.47 ± 4.31 kg). All were elderly Tunisians diagnosed with mild AD who were at least 65–80 years old, and who attended the Neurology Department of the University Hospital Center (UHC) of Monastir, Tunisia. All patients were diagnosed with AD according to the International Working Group diagnosis criteria (IGW) (Guyatt et al., 1984). Patients are diagnosed with mild-stage Alzheimer’s disease by a medical specialist and a psychophysiologist. The results of the therapeutic studies showed that the patients suffered from mild cognitive impairment and a slight problem of coordination (after cognitive and physical tests); The patients were accompanied either by their spouses, children, close friends, etc. (after cognitive and physical tests) to encourage them to get involved in the study. All were carefully screened based on the eligibility criteria cited below prior to the start of the study. Male and female participants who were able to provide written informed consent and met the eligibility criteria were included in the study.

### Eligibility criteria

All participants in this study had Mini Mental State Examination (MMSE) scores of at least 20 (MMSE: 20.82 ± 0.41), had a homogeneous education level (years: 7.6 ± 2.9) and were in the early stages of the disease. All participants presented with mild cognitive impairment. During the study, patients were always accompanied by a spouse, child, or close friend to provide encouragement and/or to facilitate their understanding of the study protocol. Only patients available to be involved in the study were selected to conduct the research. Patients who were obese with BMIs over 30, had vascular dementia by stroke, diagnosis of other types of dementia, fractures, or musculoskeletal disorders, had undergone brain surgery, showed injury or any type of fracture, who have no acceptable hearing sense, had late-stage disease, consumed drugs that may affect metabolism and the production of steroid hormones were all excluded. The study was conducted by healthcare professionals including physical therapists, occupational therapists, social workers, and researchers leading the project at different hospitals in Tunisia. All protocols were standardized throughout the duration of the study.

### Legal and ethical aspects

This study was approved by the Committee for the Protection of Persons (CPP) of Monastir, Tunisia. The purpose of the committee is to judge the scientific quality of projects, to ensure the safety of the participants, to protect their integrity, and to ensure that each subject has been able to follow experimental instructions. A further purpose was to ensure that all participants provided informed consent by signing an approved consent form; thus, the rights of human participants were protected.

### Procedures

Participants who met the criteria were given MT or PR as a single or combined intervention or served as a control group. They were divided into four groups at random. The first group, consisting of seven patients, functioned as a control group (Co). The second group, which included seven patients, took part in physical therapy (PR). The third group, which included seven patients, received music therapy (MT). The last group, which included seven patients, received both music therapy and physical rehabilitation (MT + PR). Participants engaged in the study for four months (16 weeks), with three 60-min sessions per week.

Patients with AD were treated with MT and/or PR. MT required listening to music selected by a music therapist from a repertoire of traditional Tunisian music. Musical extracts were selected by a music therapist from a repertoire of traditional Tunisian music. The selection of the extracts was made with the help of the parents of the patients who specified the preferred choices of the latter. In the majority, since the patients were of similar ages, their preferences for the majority of songs were similar and this further facilitated the study. The music was used to provide musical stimulation to patients in the MT and MT + PR groups. Music therapy that involves listening to music was found to be more successful in lowering behavioral symptoms than active music therapies in a comprehensive review and meta-analysis (Tsoi et al., 2018). Listening to music can help with verbalization, memory recall, and relaxation (Eftychios et al., 2021). Prior to beginning the study, we asked about the patients’ particular musical interests and preferences, as well as any artists or songs that may have offered some happy moments in their life. Music has a significant emotional impact, especially when it is tied to autobiographical memories. The music utilized in this study was played through speakers using personal computers. At each remediation session, the MT + PR group was always subjected to both MT and PR at the same time. Patients in the PR and MT + PR groups were given muscle strengthening exercises and joint motions tailored to their upper and lower limbs, as part of a pre-determined program (Maltais et al., 1997). 20 min of walking to improve postural parameters, muscle growth, and joint motions, 20 min of balancing and posture, and 10 min of stretching and calming the body were included in the PR. It should be emphasized that each rehabilitation session was always preceded by a 10-min warm-up training session, which consisted of walking on a treadmill at a low speed (5 km/h) to avoid muscular damage and fatigue.

### Measurements

#### Cognitive function

The evaluation of cognitive and mental function, memory capacity and intellectual aptitude of the Alzheimer’s patients was carried out prior and following the interventions using psychometric tests namely the Mini-Mental State Examination (MMSE) (Folstein et al., 1975) and ADAS-Cog Total (Rosen et al., 1984). This test was used to verify basal cognitive status, which was part of the inclusion criterion, and was also used to evaluate progression. Compared to the MMSE, the ADAS-Cog is more sensitive, reliable, and less influenced by educational level and language skills. However, it is more complex and subjective. Test-providers need not be physicians but must undergo special training prior to implementation. There is a strong association between the MMSE and ADAS-cog, indicating that where disease has been measured for one of these severity assessments, similar conclusions may be drawn for the other (Balsis et al., 2015).

**Mini Mental State Examination (MMSE):** The MMSE or Folstein test is a brief cognitive screening test which makes it possible to quickly quantify cognition capacities in the elderly including levels of cognitive and mental functions, memory capacities and intellectual abilities (Folstein et al., 1975). The test is notably used in the context of screening for dementia of the Alzheimer type. The test contains six subtests or cognitive function which explore the following areas: orientation in time and space, learning, attention and mental calculation, recall, language, and constructive praxis. The MMSE is a widely used tool because its delivery is quick (2–10 min) and requires no sophisticated materials. Age and level of education could influence the latter. It is a screening tool that makes it possible to assess the severity of the dementia, but an additional neuropsychological examination is needed for complete diagnosis. The maximum score achievable on the test is 30 points. A score above 20 suggests mild cognitive impairment in the patient. If the score is between 11 and 20, we consider a moderate cognitive impairment. Below a score of 10, the patient’s cognitive impairment is considered severe. These scores are offered in the context that the patient has already been clinically diagnosed with AD and the level of cognitive impairment has been determined (Folstein et al., 1975).

**Alzheimer’s Disease Assessment Scale-Cognitive (ADAS-Cog).** The ADAS-Cog is a scale, which makes it possible to assess the overall efficiency (memory, language and praxis), the progression or deterioration of cognitive abilities and the severity of cognitive disorders in patients with dementia of the Alzheimer type (Rosen et al., 1984). It represents a useful quantification tool for research and pharmacological trials but should not be used as a diagnostic tool. The test includes 11 sub-tests which explore the items: intelligibility of oral language, comprehension, missing the word, recall of words, naming, orientation, execution of orders, praxis, constructive praxis, word recognition and reminder of instructions. The testing usually requires several minutes depending on individual patient ability. The tests are always be presented in the same order. The scoring system reflects the degree of severity of the disorder on a scale of 0 to 5 points. Score ‘0’ corresponds to the absence of the deficit in a task. Score ‘4’ is reserved for the most severe degree of the deficit. A score of ‘1’ means a very slight deficit. Ratings ‘2’ and ‘3’ correspond respectively to a slight and moderate deficit. The total score is out of a possible 70.

#### Motor function

Each of the physical performance tests were performed twice with at least 3 min rest between trials and 15 min between tests. The order of the testing was the same for all participants as listed below. All physical measures were demonstrated to the participants before being performed. All measurements were conducted in a measuring room to exclude environment impacts.

**Gait parameters.** The walk test was carried out using the Bessou locometer (Satel, Blagnac, France). The principle of this method of data analysis is based on recording the longitudinal displacement of each foot during a walking distance of about 10 m. The movement of each foot is transmitted by a wire to an optical sensor. This information is retransmitted to a computer which displays the spatiotemporal data of the walking cycle. The acceleration and deceleration phase were removed so that the calculated values correspond to the stabilized speed (Bessou et al., 1988). Each patient was instructed to walk at a comfortable pace without gait aids on the walkway, initiating and terminating their walk 1.5 m prior and following the walkway exercise respectively. In this study, gait measurement focused on specific variable parameters of gait including the step length and walking speed.

**Six-Minute Walk Test (6MWT).** The test is a sub-maximal exercise test used to assess aerobic capacity and endurance (Pl and Sherrill, 1998). It is a safe, simple, and easy diagnostic test for evaluating motor capacity in elderly patients with cognitive and intellectual disorders (Guerra-Balic et al., 2015). The distance covered over a period of 6 min is used as the outcome measure to compare changes in performance capacity. The 6MWT consists of measuring the maximum distance covered by a subject on a hard and flat surface in 6 min without running. It allows the evaluation of the integrated response of the cardiovascular, respiratory, and muscular systems during exercise. The 6MWT was performed in a 30-meter covered corridor, the corridor was flat and straight, well-defined, and not frequented by individuals who were not involved in the study. The course outline was marked at 3-meter intervals. Two cones marked the location of the U-turns. A colored band was used to outline the starting point.

**Berg Balance Scale (BBS).** This test is a widely applied test for assessing balance in both static and dynamic situations in the elderly (Berg et al., 1992). The BBS test assesses balance and the risk of falls in relation to bodily function and activity. Since 1990, it has been the gold standard for balance. The test assesses the vestibular system, functional balance, proprioception, and strength of different activities. The BBS consists of qualitative measures of postural control capacity. It comprises 14 functional items evaluated by 5 points such as sitting to standing, standing unsupported, chair transfers, standing with eyes closed, tandem standing, and single leg standing; 0 to 4 points are given for each item, and the total score is 56. A score of 41-56 suggests a low fall risk, 21-40 a medium fall risk and 0-20 is a high fall risk. A score of 0 indicates a lack of ability to execute the task, while a score of 4 shows the achievement of the task successfully according to test criteria. A change of 8 points between two assessments indicates a clinically meaningful change in function.

#### Data analysis

For each participant, the 2 trials of the performance measures were averaged. Data are presented as mean ± standard deviation (SD). Descriptive analyses were performed to calculate the group means and standard deviations of cognitive and physical performance measures. Statistical analysis of the data as well as verification of normality was performed using the Shapiro-Wilks test due to the small number of patients. Based on a normal distribution, parametric and non-parametric tests were used. Comparisons between groups were investigated using Student’s t test or the Mann-Whitney U test. This is in accordance with the characteristics of the data. One-way repeated measures analysis of variance (ANOVA) and Bonferroni’s *post hoc* test was used to compare the effects of MT and PR as a single or combined intervention prior and post intervention. To allow a better interpretation of the results, the effect sizes were calculated as partial eta-squared (ηp^2^) for the ANOVA analysis to determine the magnitude of the change. Pearson correlation coefficient was also used to measure the linear association between the changes in step length, walking speed, 6MVT and BBS scores with changes in MMSE in the AD patients. All statistical analysis was carried out using the commercial statistical package software for social sciences (SPSS version 23.0, IBM, USA) and MedCalc Statistical software version 17.9.7 (MedCalc bvba software, Ostend, Belgium^1^; 2017). A significance level of *p* ≤ 0.05 was used for all analyses.

## Results

The study was initiated in October 2019. The study was repeated 3 times and the outcome measures were always the same. Only the results of the 3rd year have been retained. There was no dropout.

Table 1 shows the baseline cognitive status of Tunisian elderly male patients with mild AD for Co, PR, MT, and MT + PR groups. It defines their mental states assessed by the MMSE test as well as the overall efficacy measured by the ADAS-Cog test and its subscales such as memory, language, and praxis. The Mann-Whitney U test revealed no significant differences between the different groups of patients at the start of the rehabilitation program for all cognitive functions evaluated (*p* > 0.05).

**TABLE 1:** Baseline mean cognitive status ± standard deviation of Tunisian elderly male and female patients with mild AD in Co, PR, MT, and MT + PR groups (7 patients/group) after 4 months of intervention at the rate of 3 sessions of 60-min per week.

Parameters	Intervention				Significance	Severity
	**Co**	**PR**	**MT**	**MT + PR**		
MMSE	18.30 ± 0.46	18.00 ± 0.21	18.33 ± 2.61	18.22 ± 3.21	NS	Leger
Total ADAS-Cog	37.11 ± 3.03	36.89 ± 2.73	37.66 ± 5.73	37.31 ± 5.23	NS	Leger
MemoryADAS-Cog	18.22 ± 1.08	18.31 ± 0.87	18.41 ± 0.66	18.13 ± 0.93	NS	Leger
Language ADAS-Cog	14.11 ± 1.11	14.36 ± 1.14	13.96 ± 1.01	14.18 ± 0.96	NS	Leger
Orientation/praxy ADAS-Cog	03.56 ± 0.43	03.47 ± 0.66	03.71 ± 0.81	03.44 ± 0.76	NS	Leger

Intervention: Co, control; PR, physic rehabilitation; MT, music therapy; MT + PR, music therapy + physic rehabilitation.

### Effects of MT and/or PR interventions on cognitive function

**FIGURE 1:**
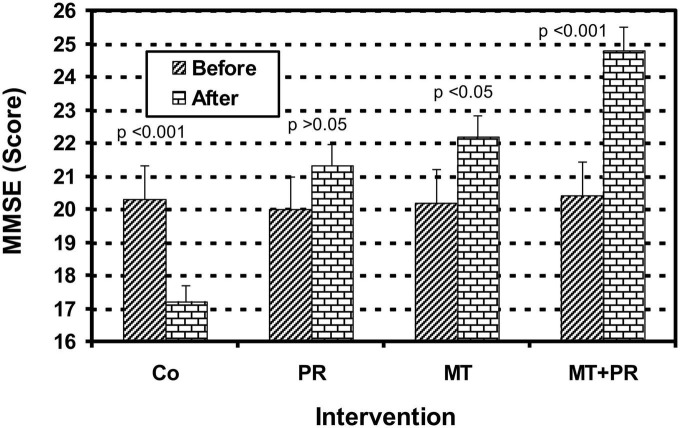
Evolution of Mini Mental State Examination (MMSE) of Tunisian elderly male patients with mild AD in Co, PR, MT, and MT + PR groups after 4 months of intervention at the rate of 3 sessions of 60-min per week. The results correspond to mean ± standard deviation for 7 patients. Intervention: Co, control; PR, physic rehabilitation; MT, music therapy; MT + PR, music therapy + physic rehabilitation.

Figure 1 shows the MMSE levels of Tunisian elderly male patients with mild AD for Co, PR, MT, and MT + PR groups after 4 months of intervention for three 60-min sessions per week. MMSE levels increased significantly in PR, MT, and MT + PR groups at the end of the intervention period, unlike the Co group (ηp^2^ = 0.56; *p* < 0.01). However, this increase was greater for MT + PR group. MT increased the cognitive index more significantly than PR (*p* < 0.05). MMSE level decreased significantly in the Co group at the end of the study (*p* < 0.001).

**FIGURE 2:**
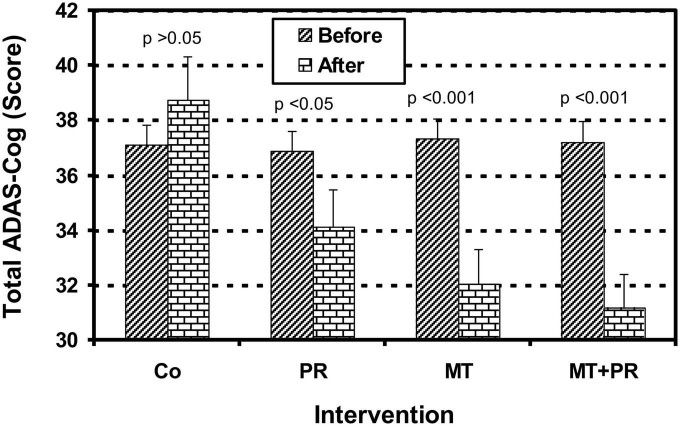
Evolution of Alzheimer’s Disease Assessment Scale Cognitive Total (ADAS-Cog Total) of Tunisian elderly male patients with mild AD in Co, PR, MT, and MT + PR groups after 4 months of intervention at the rate of 3 sessions of 60-min per week. The results correspond to mean ± standard deviation for 7 patients. Intervention: Co, control; PR, physic rehabilitation; MT, music therapy; MT + PR, music therapy + physic rehabilitation.

Figure 2 shows the ADAS-Cog Total levels of Tunisian elderly male and female patients with mild AD for Co, PR, MT, and MT + PR groups after 4 months of intervention for three 60-min sessions per week. ADAS-Cog Total levels decreased significantly for the PR, MT, and MT + PR groups at the end of the intervention period, unlike the Co group (ηp^2^ = 0,61; *p* < 0.001). However, this decrease was greater for MT + PR group. MT reduced the cognitive index more significantly than PR (*p* < 0.05). The ADAS-Cog Total level increased significantly in the Co group at the end of intervention (*p* < 0.05).

**FIGURE 3:**
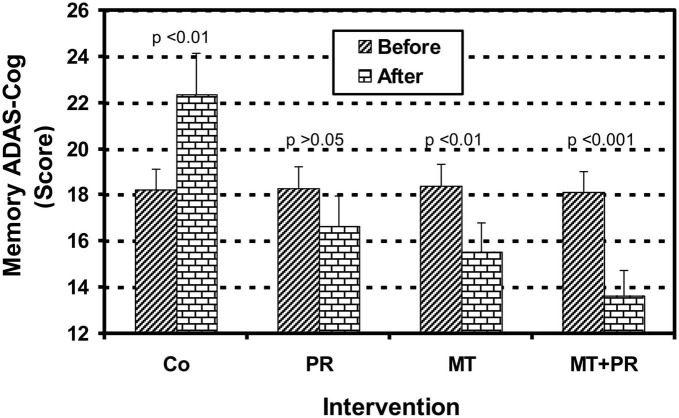
Evolution of Alzheimer’s Disease Assessment Scale Cognitive Memory (ADAS-Cog Memory)of Tunisian elderly male patients with mild AD in Co, PR, MT, and MT + PR groups after 4 months of intervention at the rate of 3 sessions of 60-min per week. The results correspond to mean ± standard deviation for 7 patients. Intervention: Co, control; PR, physic rehabilitation; MT, music therapy; MT + PR, music therapy + physic rehabilitation.

Figure 3 shows the ADAS-Cog Memory levels of Tunisian elderly male and female patients with mild AD for Co, PR, MT, and MT + PR groups after 4 months of intervention for three 60-min sessions per week. ADAS-Cog Memory levels decrease significantly for the PR, MT, and MT + PR groups at the end of the intervention period, unlike the Co group (ηp^2^ = 0.61; *p* < 0.01). However, this decrease was greater for the MT + PR group. MT reduced the cognitive index more significantly than PR (*p* < 0.05). The ADAS-Cog Memory level increased significantly in the Co group at the end of intervention as recorded by the Wilcoxon Signed Rank test (*p* < 0.01).

### Effects of MT and/or PR on motor function

**FIGURE 4:**
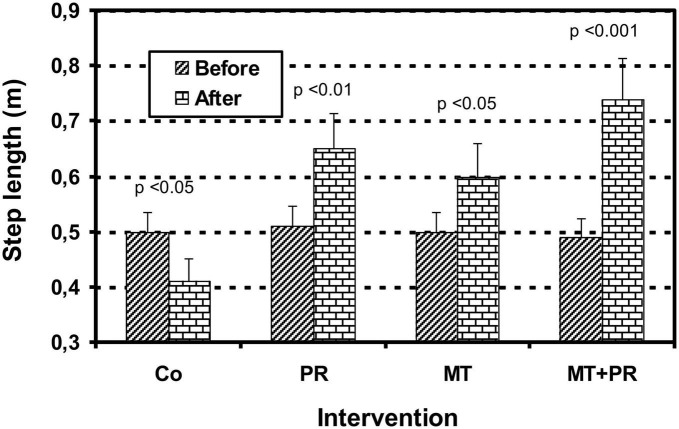
Evolution of step length (m) of Tunisian elderly male patients with mild AD in Co, PR, MT, and MT + PR groups after 4 months of intervention at the rate of 3 sessions of 60-min per week. The results correspond to mean ± standard deviation for 7 patients. Intervention: Co, control; PR, physic rehabilitation; MT, music therapy; MT + PR, music therapy + physic rehabilitation.

Figure 4 shows the evolution of step length levels of Tunisian elderly male and female patients with mild AD for Co, PR, MT, and MT + PR groups after 4 months of intervention for three 60-min sessions per week. Step length increased significantly in the PR, MT, and MT + PR groups at the end of the intervention period, unlike the Co group (ηp^2^ = 0.63; *p* < 0.001). However, this increase was greater for MT + PR group. PR increased step length more than MT (*p* < 0.05). The step length decreased significantly in the Co group at the end of the intervention (*p* < 0.05). Changes in MMSE scores were positively correlated in the PR, MT, and MT + PR groups with changes in the step length levels (*r* = 0.77; *p* < 0.001).

**FIGURE 5:**
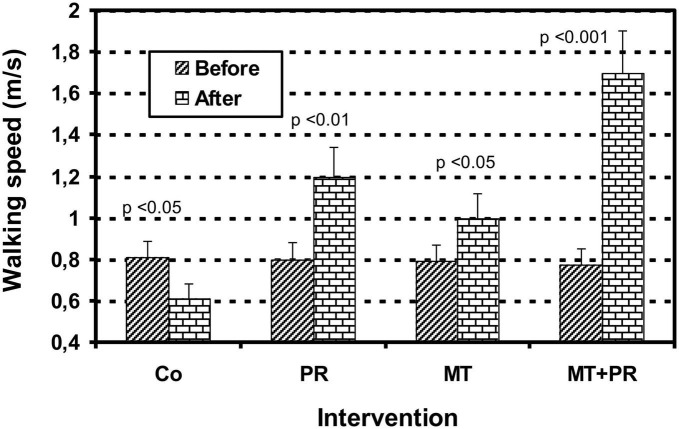
Evolution of walking speed (m/s) of Tunisian elderly male patients with mild AD in Co, PR, MT, and MT + PR groups after 4 months of intervention at the rate of 3 sessions of 60-min per week. The results correspond to mean ± standard deviation for 7 patients. Intervention: Co, control; PR, physic rehabilitation; MT, music therapy; MT + PR, music therapy + physic rehabilitation.

Figure 5 shows the evolution of walking speed levels of Tunisian elderly male and female patients with mild AD for Co, PR, MT, and MT + PR groups after 4 months of intervention for three 60-min sessions per week. Walking speed increased significantly in the PR, MT, and MT + PR groups at the end of the intervention period, unlike the Co group (ηp^2^ = 0.59; *p* < 0.001). However, this increase was greater for MT + PR group. PR increased walking speed more than MT (*p* < 0.05). The walking speed decreased significantly in the Co group at the end of the intervention (*p* < 0.05). Changes in MMSE scores were positively correlated in the PR, MT, and MT + PR groups with changes in the walking speed levels (*r* = 0.73; *p* < 0.001).

**FIGURE 6:**
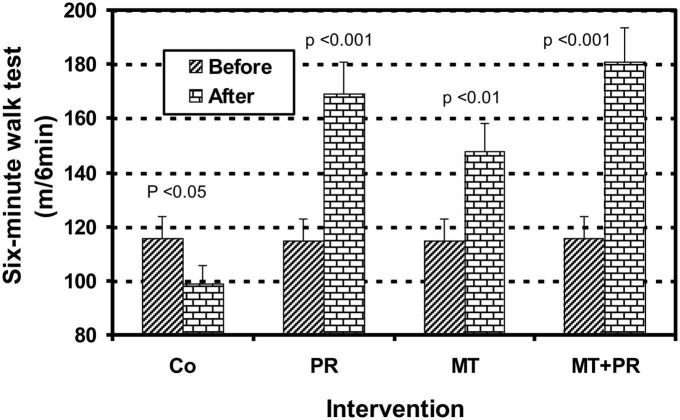
Evolution of distance covered in 6 min evaluated by the 6 Minute Walk Test (6MWT) walking speed (m/s) of Tunisian elderly male patients with mild AD in Co, PR, MT, and MT + PR groups after 4 months of intervention at the rate of 3 sessions of 60-min per week. The results correspond to mean ± standard deviation for 7 patients. Intervention: Co, control; PR, physic rehabilitation; MT, music therapy; MT + PR, music therapy + physic rehabilitation.

The 6MWT consists of evaluating the maximum distance achieved by a patient on a flat and hard surface after 6 min of walking. Figure 6 illustrates the evolution of the distance covered in 6 min by Tunisian elderly male and female patients with mild AD for Co, PR, MT, and MT + PR groups after 4 months of intervention for three 60-min sessions per week. The distance carried out in 6 min increase significantly in the PR, MT, and MT + PR groups after the intervention period, unlike the Co group (ηp^2^ = 0.61; *p* < 0.001). However, the distance covered over a time of 6 min was greater for MT + PR group. Patients in PR group walked a longer distance in comparison with the MT group (*p* < 0.05). In the Co, the distance walked in 6 min decreased significantly at the end of the intervention (*p* < 0.05). Changes in MMSE scores were positively correlated in the PR, MT, and MT + PR groups with changes in the 6MVT levels (*r* = 0.75; *p* < 0.001).

The BBS test allows the evaluation of stability and the risk of falls in elderly people who have functional balance disorders.

**FIGURE 7:**
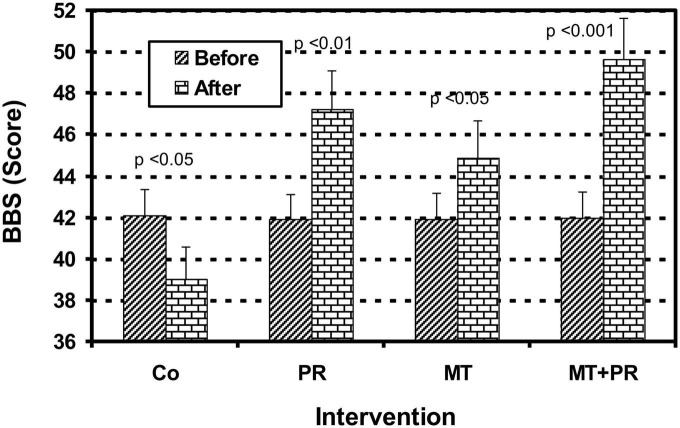
Evolution of postural balance evaluated by the Berg Balance Scale (BBS) of Tunisian elderly male patients with mild AD in Co, PR, MT, and MT + PR groups after 4 months of intervention at the rate of 3 sessions of 60-min per week. The results correspond to mean ± standard deviation for 7 patients. Intervention: Co, control; PR, physic rehabilitation; MT, music therapy; MT + PR, music therapy + physic rehabilitation.

Figure 7 shows the balance index of Tunisian elderly male and female patients with mild AD for Co, PR, MT, and MT + PR groups after 4 months of intervention for three 60-min sessions per week. Static and dynamic balance abilities in patients with AD improved significantly in the PR, MT, and MT + PR groups after the intervention period, unlike the Co group (ηp^2^ = 0.59; *p* < 0.001). However, this increase was greater for MT + PR group. PR increased performance in BBS more than MT (*p* < 0.05). The performance in BBS decreased significantly in the Co group at the end of the intervention (*p* < 0.05). Changes in MMSE were positively correlated in the PR, MT, and MT + PR groups with changes in the BBS score (*r* = 0.78; *p* < 0.001).

## Discussion

In its typical form, AD is characterized by impaired memory of recent events, unusual, repeated forgetfulness and difficulty learning and retaining new information (Grush, 2006). The evolution of the disease is accompanied by impairment of cognitive domains, in particular language or aphasia, perception or agnosia, and gestural ability or apraxia. It is characterized by the progressive and irreversible degeneration of nerve cells. The disappearance of the latter leads to the continuous decline of cognitive capacities formerly called intellectual capacities (Cantegreil-Kallen et al., 2005; Calabrò et al., 2021). AD is often associated with memory loss because the neurons located in the region of the hippocampus, the seat of memory are the first to be affected. Other areas of the brain eventually become susceptible to the condition leading to the alteration of orientation abilities in time and space, recognition of objects and people, use of language, reasoning, balance, and posture (Morris et al., 1987; Cantegreil-Kallen et al., 2005). These disorders gradually reduce autonomy. Indeed, it has been demonstrated that when certain pathological processes affect the central nervous system, there appear to be fluctuations in gait parameters associated with the risk of falls. On the other hand, discordances in the gait of people with dementia cause a reduction in mobility as well as an increase in falls (Morris et al., 1987; Van Doorn et al., 2003). The risk of falls is doubly increased in the elderly with dementia (Van Doorn et al., 2003; Calabrò et al., 2021).

Our study is part of an ongoing experimental investigation that aims to assess the impact of non-drug treatments for AD. Our objective was to examine the effectiveness of a non-drug regime combining a cognitive stimulation program based on MT with a PR program on cognitive and motor function in elderly Tunisian male and female patients with mild AD. We designed this study examining cognitive and motor functioning because these measures represent the most widely used outcome markers in studies of non-drug therapies in AD. Indeed, studies investigating the impact of cognitive stimulation and physical intervention in patients with AD generally focus on general cognitive functioning and typically use the MMSE and ADAS-Cog as assessment tools (Olazarán et al., 2004). The evaluation of postural balance, risk of falls, walking speed, step length and distance covered in 6 min are also indices for evaluating cognitive decline in AD (Larson et al., 2006). Our study demonstrated that MT and PR used as a single intervention significantly improved in the cognition indices MMSE, ADAS-Cog Total and the ADAS-Cog Memory subscale. This was done by increasing respectively the first measure and reducing the last two total measurements in the cognition indices used. Further to this, there were increases in the length of the step, walking speed, the distance covered in 6 min and postural balance in static and dynamic situations evaluated by the BBS scale. The study also demonstrated that the combination of these two intervention methods (MT + PR) improved the indices more than the interventions in isolation. Our study also demonstrated that MT improves more effectively cognitive function while PR has a greater effect on motor function. This shows that these two non-drug rehabilitation techniques could provide therapeutic potential for the improvement of multiple aspects of cognitive and motor functions in elderly Tunisian adults male and female with mild AD. These results agree with the findings obtained by Palleschi et al. (1996), Rolland et al. (2007), and Chortane et al. (2022) who reported that endurance exercise improved global cognitive functions assessed using the MMSE, and walking and balance in people with AD. Also, Shen et al. confirmed that a physical activity program significantly improves muscle strength, flexibility, agility, dynamic balance, endurance, walking and decreases the risk of falls following a program of 12 weeks (Shen et al., 2008). The interest of a physical exercise program in improving the spatio-temporal parameters of walking and balance in the elderly has also been reported (Shigematsu, 2002; Sherrington et al., 2008; Thomas et al., 2010).

Although data on the effects of cognitive reduction on the control of AD remain rare, some studies agree with our results, and have reported positive effects of cognitive stimulation on cognitive decline in AD. Indeed, it has also been reported improved mood, episodic memory, language, attention, and executive functions in Alzheimer’s patients who were subjected to MT (Särkämö et al., 2015). Musical memo therapy is a therapeutic approach to AD symptoms and has been used effectively for the reminiscence of memories and episodes and the recall of emotionally rich autobiographical events in some patients with AD (Van de Winckel et al., 2004; Eftychios et al., 2021), also showed that music allows the recovery of functions such as memory and language as well as the ability to recall past events in AD patients. Singing workshops have also shown that patients with AD retain learning ability. When taught new songs, these patients manage to memorize the melody of the song presented to them. Another study also pointed out that significant improvements are noted on anxiety and depression in patients who have benefited from therapy using music and the effects have even been maintained for several weeks following the cessation of therapy (Guetin et al., 2013). The musical memory of patients with AD seems to be relatively preserved during its evolution for patients who were musicians before (Groussard et al., 2013).

ADAS and MMSE are widely used screening tools that provide a quantitative assessment of the cognitive conditions of the Alzheimer’s patients in both routine clinical practice and research settings, and the results obtained by one or the other in the clinical diagnosis of AD are always strongly negatively correlated (Lombardi et al., 2020). For this reason, we contented ourselves in this study by establishing the relationship between cognitive and motor functions only by only using the MMSE tool. By increasing total level in Alzheimer’s patients’ in the PR, MT and MT + PR groups, variations in MMSE scores were correlated positively with improvements in all motor functions scores assessed in this study such as step length, walking speed, 6MVT, and the BBS scores. This shows that cognitive and motor performances are strongly associated in AD and that the MMSE is a reliable tool for establishing the relationship between these two functions. The significant association between change in motor function and cognition in older adults with AD has been reported (Hesseberg et al., 2020). Motor function impairment because of cognitive impairment is not uncommon (Liou et al., 2020). Indeed, a lower cognitive function level is a risk factor for the development of motor impairment, especially falls and a more rapid rate of motor decline (Hausdorff and Buchman, 2013). Conversely, a lower motor function level, such as slow gait, is also a risk factor for the development of mild cognitive impairment, dementia, and a more rapid rate of cognitive decline (Inzitari et al., 2007; Hausdorff and Buchman, 2013). The mechanism underlying the relationship between motor performance and cognitive functions is not well understood. Researchers used to believe that cognition and motor functions developed separately. However, recent studies have demonstrated that motor and cognitive function develop along a similar trajectory (Liou et al., 2020). Some studies have reported that motor and cognitive processes share a similar evolutionary history, and some brain regions integrate both motor and cognitive functions (Lee, 2020). This theory would certainly support the findings of the present study. This theory probably supports the relationships observed in elderly Tunisian male and female patients with mild Alzheimer’s patients between the improvement of cognitive and motor functions following the PR, MT, and MT + PR interventions.

The main limitation of this study relies on the fact that these data are specific to Tunisian music and participants from Tunisia. Moreover, the number of effective participants in this study was small 26. However, we did find significant differences between groups. As all the participants were accompanied by their family members so family encouragement which can account for improvements in cognition and motor performance which should be taken in consideration in future studies. To confirm the findings reported here, large scale experimental measurements are needed with greater subject numbers.

## Conclusion

In conclusion, this study highlights the importance of a combination of MT and PR interventions in the improvement of cognitive and motor functions in elderly male and female Tunisian patients with mild AD. Furthermore, intervention for 4 months (3 times/week) for 60 min gave favorable improvement of cognitive and motor functions in MT, PR and MT + PR group compared to Co. The cognitive as well as motor functions improvement in MT + PR group were more favorable compared to MT or PR group individually. Interestingly, MT group showed favorable improvements in cognitive whereas PR group showed favorable improvements in motor functions among Tunisian population.

## Data availability statement

The raw data supporting the conclusions of this article will be made available by the authors, without undue reservation.

## Ethics statement

The studies involving human participants were reviewed and approved by Committee for the Protection of Persons (CPP) of the Monastir region, Tunisia. The patients/participants provided their written informed consent to participate in this study.

## Author contributions

SC, CC, and TG wrote the manuscript. MB and NB helped in data analysis. KS, AS, and DT helped in figures and revising the manuscript. All authors contributed to the article and approved the submitted version.

## Footnotes

1
http://www.medcalc.org
